# Construction and Identification of Eukaryotic Expression Vector pEGFP-N1-MIC-1 for Mouse MIC-1 Gene and Its Effect on Gastric Cancer Cells

**DOI:** 10.1155/2024/2165242

**Published:** 2024-07-16

**Authors:** HuiPeng Zhang, Zhongyu Qin, ShuaiShuai Shi, YunFei Li, Yang Song, YiQiang Zhang

**Affiliations:** ^1^ Department of General Surgery Heji Hospital Affiliated to Changzhi Medical College, Changzhi, Shanxi 046011, China; ^2^ Department of Basic Medicine Changzhi Medical College, Changzhi, Shanxi 046000, China; ^3^ Department of Nephrology Heji Hospital Affiliated to Changzhi Medical College, Changzhi, Shanxi 046011, China; ^4^ Institute of First Clinical Changzhi Medical College, Changzhi, Shanxi 046000, China; ^5^ Laboratory Animal Center (LAC) Changzhi Medical College, Changzhi, Shanxi 046000, China

## Abstract

This study aimed to construct an eukaryotic expression vector, pEGFP-N1-MIC-1, for overexpressing the mouse macrophage inhibitory cytokine-1 (MIC-1) gene. Additionally, we transfected the MFC cell line to observe the upregulation of MIC-1 gene expression and assess its impact on macrophage phenotype conversion. Enzyme digestion and DNA sequencing confirmed the successful construction of the pEGFP-N1-MIC-1 vector. The transfected MFC cells exhibited a significant increase in MIC-1 protein expression levels. Furthermore, transfection with pEGFP-N1-MIC-1 increased the migration and colony formation capabilities of MFC cells. These results may contribute to future research and the development of therapeutic interventions targeting MIC-1 in macrophages, particularly in the context of gastric cancer.

## 1. Introduction

Gastric cancer, a malignant tumor affecting the digestive tract, poses a significant threat to individuals, families, and communities, with a 5-year survival rate of less than 40% [1]. Currently, effective treatments for gastric cancer are lacking, necessitating a multidisciplinary approach to diagnosis and treatment. Therefore, it is crucial to investigate the mechanisms underlying gastric cancer development and prevention.

Macrophage inhibitory cytokine-1 (MIC-1), a vital member of the transforming growth factor-*β* superfamily, was initially discovered by Bootcov et al. [2] and isolated from the cDNA library of U937 macrophages. MIC-1 plays a pivotal role in the development of various digestive system tumors [3]. In early stages of gastric cancer, MIC-1 induces apoptosis and inhibits proliferation, demonstrating anticancer effects. However, in the advanced stage of gastric cancer, changes in the internal environment lead to a pro-cancer effect of MIC-1. These effects are potentially mediated through various mechanisms, including inhibition of *β*-catenin gene expression, increased protease activity, enhanced degradation of stromal collagen, and decreased cell adhesion. Ultimately, these changes result in cancer cell detachment and metastasis [4].

To further investigate the regulatory role of MIC-1 in gastric cancer development, this study aimed to construct an eukaryotic expression vector, pEGFP-N1-MIC-1, for the MIC-1 gene. The successful construction of this vector will provide a solid foundation for future research endeavors.

## 2. Materials and Methods

### 2.1. Cell Culture and Treatment

The MFC cells were cultured in RPMI-1640 (01-100-1A, BI, Kibbutz Beit Haemek, Israel) medium supplemented with 10% fetal bovine serum (FBS; 04-001-1A, BI, Kibbutz Beit Haemek, Israel) and antibiotics at 37°C with 95% humidity and 5% CO_2_. The cells were seeded at a density of 1 × 10^6^ cells/well in a 6-well plate. Transfection was performed using Lipofectamine TM 2000 (Invitrogen, Carlsbad, CA, USA) according to the manufacturer's instructions. After 24 hr, the cells were divided into control and pEGFP-N1-MIC-1 groups. The transfection procedure involved separately dissolving the recombinant plasmid pEGFP-N1-MIC-1 and Lipofectamine TM 2000 in serum-free RPMI-1640 culture medium, followed by mixing and incubation. The plasmid–lipid mixture was added to the 6-well plate and incubated at 37°C with 5% CO_2_ for 6 hr. After that, the medium was replaced with normal culture medium containing serum without antibiotics, and the cells were cultured for another 24 hr. Cell growth and enhanced green fluorescent protein (EGFP) expression were observed using a fluorescence microscope.

### 2.2. Extraction and Reverse Transcription of Mouse Gastric Tissue RNA and PCR Amplification of MIC-1 Gene Sequence

Healthy mouse gastric tissue was isolated, and total RNA was extracted using Trizol (9108). RNA integrity was assessed using a 1% agarose gel. The intact RNA was reverse transcribed into cDNA using 5x Primescript RT Master Mix. The MIC-1 gene sequence was obtained from the GenBank database, and primers for MIC-1 were designed with QuickCut HindIII (1,615) and QuickCut EcoRI (1,605) restriction endonuclease sites in the forward and reverse primers, respectively. The PCR reaction mixture contained GoTaq qPCR Master Mix (M722), forward and reverse primers, MIC-1 DNA, and sterile water. The resulting PCR products were analyzed by agarose gel electrophoresis.

### 2.3. Construction and Detection of Recombinant Plasmid pMD18T-MIC-1

PCR products, pMD-18T vector, and ligation solution were mixed for ligation and transformed into competent *E. coli* DH5*α*. Plasmid extraction was performed on the resulting bacterial culture, and QuickCut HindIII and QuickCut EcoRI restriction enzymes were used for plasmid digestion. The digestion products were analyzed by agarose gel electrophoresis, and the plasmid with correct restriction patterns was sent for sequencing analysis.

### 2.4. Construction and Detection of pEGFP-N1-MIC-1 Plasmid

Fragments of pMD18T-MIC-1 plasmid with correct sequencing were cut out from the agarose gel using QuickCut HindIII and QuickCut EcoRI enzymes and then recovered using a gel recovery kit. Large fragments of pEGFP-N1 plasmid were cut with the same enzymes, and the recovered fragments were used for ligation. The ligation product was sent for sequencing analysis to confirm the correctness of the final recombinant plasmid, named pEGFP-N1-MIC-1.

### 2.5. Cell Transfection

MFC cells were seeded in a 6-well plate and transfected with the pEGFP-N1-MIC-1 plasmid using Lipofectamine TM 2000. After 6 hr, the medium was replaced, and the cells were cultured for an additional 24 hr. Transfection efficiency was evaluated by monitoring cell growth and EGFP expression under a fluorescence microscope.

### 2.6. Western Blotting to Identify MIC-1 Protein Expression

Total protein was extracted from transfected cells and subjected to western blot analysis using an anti-FLAG-Tag antibody (RG001060). The expression of MIC-1 protein was observed using chemiluminescence.

### 2.7. Cell Cycle Analysis

After 48 hr of cell transfection, cells were collected by digestion with trypsin, washed twice with PBS, and the supernatant was discarded. Then, 1 ml of 70% precooled ethanol was added to the cells, and they were gently vortexed and fixed at 4°C for at least 24 hr. After that, the cells were washed twice with PBS and resuspended in 0.5 ml of PBS. Then, propidium iodide (PI, Solarbio, IP5030) and RNaseA (R8023) were added to a final concentration of 50 *µ*g/ml, and the cells were incubated at 37°C for 30 min. Finally, cell cycle analysis was performed using flow cytometry. PI, propidium iodide, was prepared at a concentration of 1 mg/ml in PBS and stored at 4°C. RNase A was prepared at a concentration of 10 mg/ml.

### 2.8. Cell Migration Assay

The migratory ability of MFC cells after pEGFP-N1-MIC-1 transfection was detected by using wound healing assay. Briefly, transfected cells were seeded onto a 6-well plate until they reached 90% confluence. Subsequently, the cell layers of the treated and control groups were uniformly scratched, and the floating cells were washed with sterile PBS and replaced with serum-free medium. The migration of cells in the two groups was observed under a microscope at 24 hr, and the change in the migration ability of both groups was analyzed using the ImageJ software.

### 2.9. Colony Formation Assay

To further observe its proliferative ability, after transfection for 48 hr, we seeded the cells onto a 6-well plate. The cells were cultured in a medium containing 10% FBS for 2 weeks, with medium change every 3 days until visible colonies grew. Subsequently, the cells were washed once with 1 ml of PBS to remove the supernatant, fixed with 1 ml of methanol, and stained with crystal violet. Colonies containing more than 40 cells were counted, and the results were analyzed using ImageJ software.

## 3. Results

### 3.1. Mouse Gastric Tissue RNA Extraction

**Figure 1:**
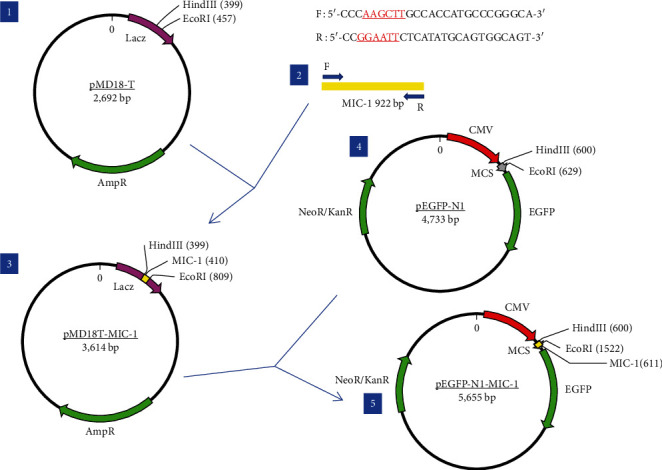
Diagram of the vector construction.

**Figure 2:**
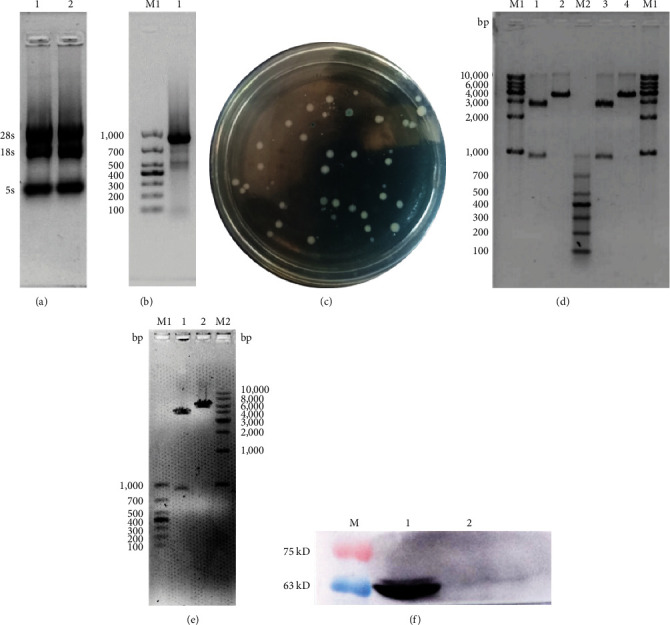
(a) Mouse gastric tissue RNA extraction results. Lanes 1 and 2 showed extracted RNA, (b) MIC-1 gene amplification image. Lane M1 showed DL1000 DNA Ladder, Lane 1 showed RT-PCR amplified MIC-1 gene fragment, (c) This figure showed white colonies were positive bacteria, and blue colonies were negative bacteria, (d) This figure showed pMD18-T-MIC-1 digestion figure. Lane M1 showed 1 kb DNA Ladder, Lane M2 showed DL1000 DNA Ladder, Lanes 1,3 showed QuickCut HindIII and QuickCut EcoRI double digestion of pMD18-T-MIC-1, with a large fragment of 2,692 bp and a small fragment of 922 bp, Lanes 2,4: showed single enzyme digestion of pMD18-T-MIC-1 with QuickCut HindIII, with a band size of ~3,614 bp, (e) Lane M1 showed DL1000 DNA Ladder, Lane M2 showed 1 kb DNA Ladder; Lane 1 showed QuickCut HindIII and QuickCut EcoRI enzyme digestion pEGFP-N1-MIC1 results; Lane 2 showed pEGFP-N1-MIC1 single enzyme digestion results, and (f) Lane M showed protein marker, Lane 1 showed pEGFP-N1-MIC1 group, Lane 2 showed pEGFP-N1 group.

The vector construction process is illustrated in Figure 1. PCR amplification demonstrated that the amplified MIC-1 target fragment aligned with the theoretical result of 922 bp (Figure 2(a)). The purified and recovered PCR-amplified MIC-1 gene fragment was ligated with the pMD18-T vector, resulting in visible positive colonies after transformation (Figure 2(c)). The positive recombinant plasmid was named pMD18-T-MIC-1 following further extraction and digestion with QuickCut HindIII and QuickCut EcoRI. Gel analysis revealed two bands, representing the pMD18-T vector and the 922 bp target fragment, confirming the correct plasmid connection (Figure 2(d)). The pEGFP-N1-MIC-1 vector was digested with QuickCut HindIII and QuickCut EcoRI, resulting in bands that matched the expected 922 bp target fragment and a large fragment of 4.7 kb. Single digestion with QuickCut EcoRI generated a band between 5,000–6,000 bp, consistent with the expected result (Figure 2(e)). MIC-1 protein expression was observed in the pEGFP-N1-MIC-1 group, with a molecular weight of ~34 kD. The EGFP exhibited a molecular weight of 27 kD, while the fusion protein displayed a molecular weight of ~61 kD. The target protein closely matched the theoretical value of 63 kD. These results confirmed successful MIC-1 protein expression in MFC cells transfected with the pEGFP-N1-MIC-1 plasmid (Figure 2(f)).

### 3.2. Flow Cytometry Analysis of Cell Cycle

**Figure 3:**
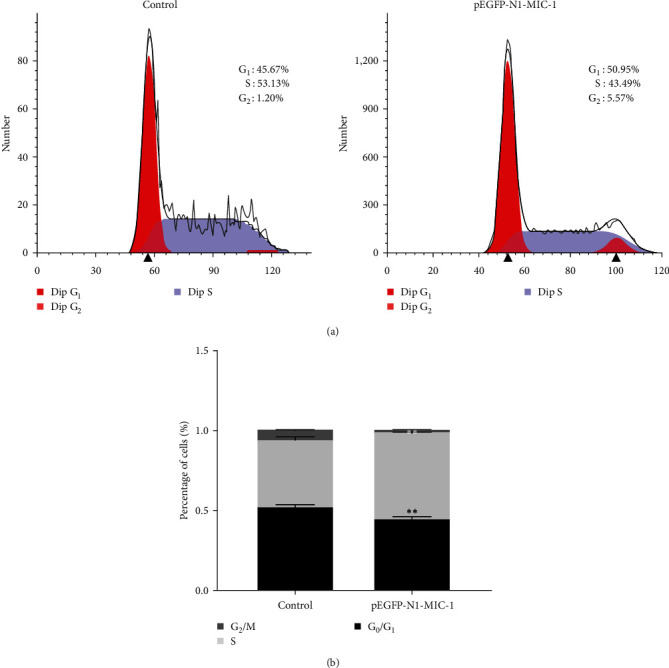
(a and b) pEGFP-N1-MIC1 effectively facilitates cell cycle progression at the G0/G1 phase and increases the number of cells entering the S phase. The difference in results is statistically significant ( ^*∗∗*^*p* < 0.01) compared to the NC group.

Compared to the NC group, the MFC cells in the pEGFP-N1-MIC-1 group exhibited a decrease in the proportion of G0/G1 phase cells and an increase in the number of cells entering the S phase (Figure 3;  ^*∗∗*^*p* < 0.01).

### 3.3. Cell Migration Assay

**Figure 4:**
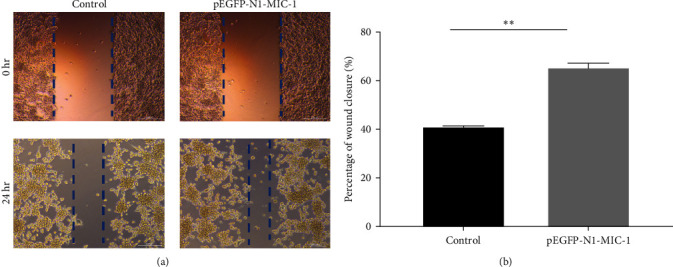
(a) The effect of pEGFP-N1-MIC1 on cell migration was assessed using wound healing assays (200x). (b) Results from a representative experiment, with triplicates, indicate that pEGFP-N1-MIC1 significantly facilitated cell migration, as demonstrated by the calculated mean values and standard deviation ( ^*∗∗*^*p* < 0.01).

To evaluate the impact of MIC-1 on the migration capability of MFC gastric cancer cells, cell scratch assays were performed. Remarkably, overexpression of the MIC-1 gene significantly enhanced the migration ability of MFC cells. Quantitative analysis at 24 hr using TScratch software revealed that MFC cells with MIC-1 gene overexpression exhibited stronger scratch healing ability compared to the control group (Figure 4;  ^*∗∗*^*p* < 0.01). These findings suggest that inhibition of the MIC-1 gene negatively regulates the migration of MFC cells.

### 3.4. Colony Formation Assay

**Figure 5:**
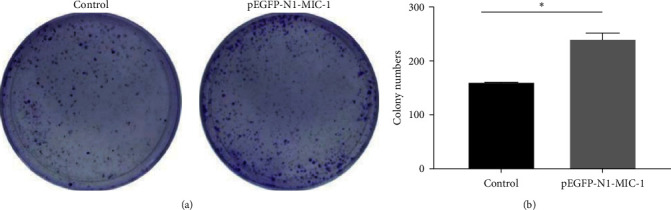
(a) The effect of pEGFP-N1-MIC1 on cell proliferation was assessed using colony formation assays (200x). (b) Results from a representative experiment, with triplicates, indicate that pEGFP-N1-MIC1 significantly facilitated cell proliferation, as demonstrated by the calculated mean values and standard deviation ( ^*∗*^*p* < 0.05).

The results demonstrated that the pEGFP-N1-MIC-1 transfected group displayed an increased number of colony-forming cells compared to the control group (Figure 5;  ^*∗*^*p* < 0.05). These findings suggest that MIC-1 expression is associated with proliferation of colony formation by MFC cells.

## 4. Discussion

The occurrence and development of gastric cancer is a continuous and complex process [5]. With advancements and profound research methods, researchers have explored the mechanisms of gastric cancer occurrence and metastasis from various perspectives and made significant breakthroughs. These factors can be broadly classified into four categories, including environment, infection, genomic DNA level, and epigenetics [6]. All these factors play a role in multiple stages of gastric cancer progression, interact with each other, and collectively promote the occurrence and metastasis of gastric cancer.

MIC-1 belongs to the transforming growth factor-*β* superfamily. It is secreted by active macrophages and has a dual role of inhibition and promotion, participating in multiple stress responses [7, 8]. MIC-1 is expressed to varying degrees in placental, digestive tract, nervous system, and prostate epithelial tissues. Studies have found high expression levels of MIC-1 in various cancer cells and tissues, such as esophageal cancer, lung cancer, prostate cancer, colon cancer, and pancreatic cancer [9, 10, 11]. Its expression level is closely related to the formation, occurrence, and development of tumors, and it participates in tumor cell proliferation, invasion, and metastasis processes [12, 13, 14]. Given its dual role of inhibition and promotion, this study constructed a pEGFP-N1-MIC-1 eukaryotic expression vector by amplifying the MIC-1 gene. Enzymatic cleavage and sequencing analysis confirmed the successful construction of the vector, which was then transferred into MFC cells. Increased EGFP expression was observed under fluorescence microscopy, and western blot analysis showed a significant increase in MIC-1 protein expression, indicating the successful construction of the MIC-1 overexpression cell line and laying the foundation for subsequent studies.

There is evidence indicating that MIC-1 has both carcinogenic and anticancer effects [15]. Literature reports suggest that MIC-1 plays an antitumor role and acts as a negative growth factor in the early stages of cancer [16]. Its activation region is targeted by the tumor suppressor gene p53, which can induce an increase in MIC-1 expression by causing cell cycle arrest and apoptosis [17]. Some studies have also demonstrated that in the middle and late stages of cancer, as the tumor microenvironment changes, MIC-1 expression increases with cancer progression, depth of invasion, and lymph node metastasis, encompassing the entire process of tumor progression [18, 19].

The dual biological roles of MIC-1 in promoting and inhibiting tumors still require further research. Previous studies have found a direct correlation between MIC-1 levels in the serum of gastric cancer patients and the progression of gastric cancer [20]. However, the mechanism of MIC-1 in the occurrence and development of gastric cancer remains unclear. Our study aims to further investigate the biological functions of MIC-1 and its related mechanisms in the occurrence and development of gastric cancer using the MIC-1 overexpression cell line.

## 5. Conclusions

The MIC-1 indeed exerts an impact on the cellular proliferation of gastric cancer cells while promoting their migration and invasive abilities, potentially leading to further deterioration of gastric cancer. These findings hold certain prognostic significance for the clinical management of gastric cancer.

## Data Availability

All research data are documented in the submitted files.

## References

[1] Liu B., Li X., Sun F. (2020). HP-CagA+ regulates the expression of CDK4/CyclinD1 via *reg3* to change cell cycle and promote cell proliferation. *International Journal of Molecular Sciences*.

[2] Bootcov M. R., Bauskin A. R., Valenzuela S. M. (1997). MIC-1, a novel macrophage inhibitory cytokine, is a divergent member of the TGF-*β* superfamily. *Proceedings of the National Academy of Sciences*.

[3] Sugimoto M., Takagi T., Konno N. (2017). The efficacy of biliary and serum macrophage inhibitory cytokine-1 for diagnosing biliary tract cancer. *Scientific Reports*.

[4] Liu T., Bauskin A. R., Zaunders J. (2003). Macrophage inhibitory cytokine 1 reduces cell adhesion and induces apoptosis in prostate cancer cells. *Cancer Research*.

[5] Smyth E. C., Nilsson M., Grabsch H. I., van Grieken N. C. T., Lordick F. (2020). Gastric cancer. *The Lancet*.

[6] Machlowska J., Baj J., Sitarz M., Maciejewski R., Sitarz R. (2020). Gastric cancer: epidemiology, risk factors, classification, genomic characteristics and treatment strategies. *International Journal of Molecular Sciences*.

[7] Siddiqui J. A., Pothuraju R., Khan P. (2022). Pathophysiological role of growth differentiation factor 15 (GDF15) in obesity, cancer, and cachexia. *Cytokine & Growth Factor Reviews*.

[8] Wischhusen J., Melero I., Fridman W. H. (2020). Growth/differentiation factor-15 (GDF-15): from biomarker to novel targetable immune checkpoint. *Frontiers in Immunology*.

[9] Brown D. A., Hance K. W., Rogers C. J. (2012). Serum macrophage inhibitory cytokine-1 (MIC-1/GDF15): a potential screening tool for the prevention of colon cancer?. *Cancer Epidemiology, Biomarkers & Prevention*.

[10] Kaur S., Chakraborty S., Baine M. J. (2013). Potentials of plasma NGAL and MIC-1 as biomarker(s) in the diagnosis of lethal pancreatic cancer. *PLoS ONE*.

[11] Xu C., Li L., Wang W., Zhang Q., Zhang X., Yang R. (2021). Serum macrophage inhibitory cytokine-1 as a clinical marker for non–small cell lung cancer. *Journal of Cellular and Molecular Medicine*.

[12] Danta M., Barber D. A., Zhang H. P. (2017). Macrophage inhibitory cytokine-1/growth differentiation factor-15 as a predictor of colonic neoplasia. *Alimentary Pharmacology & Therapeutics*.

[13] Fisher O. M., Levert-Mignon A. J., Lord S. J. (2015). MIC-1/GDF15 in Barrett’s oesophagus and oesophageal adenocarcinoma. *British Journal of Cancer*.

[14] Liu Y.-N., Wang X.-B., Wang T. (2016). Macrophage inhibitory cytokine-1 as a novel diagnostic and prognostic biomarker in stage I and II nonsmall cell lung cancer. *Chinese Medical Journal*.

[15] Song M., Mehta R. S., Wu K. (2016). Plasma inflammatory markers and risk of advanced colorectal adenoma in women. *Cancer Prevention Research*.

[16] Gkretsi V., Stylianou A., Kalli M. (2020). Silencing of growth differentiation factor-15 promotes breast cancer cell invasion by down-regulating focal adhesion genes. *Anticancer Research*.

[17] Hazafa A., Iqbal M. O., Javaid U. (2022). Inhibitory effect of polyphenols (phenolic acids, lignans, and stilbenes) on cancer by regulating signal transduction pathways: a review. *Clinical and Translational Oncology*.

[18] Bauskin A. R., Brown D. A., Kuffner T. (2006). Role of macrophage inhibitory cytokine-1 in tumorigenesis and diagnosis of cancer. *Cancer Research*.

[19] Huang M., Narita S., Koizumi A. (2021). Macrophage inhibitory cytokine-1 induced by a high-fat diet promotes prostate cancer progression by stimulating tumor-promoting cytokine production from tumor stromal cells. *Cancer Communications*.

[20] Wang Y., Jiang T., Jiang M., Gu S. (2019). Appraising growth differentiation factor 15 as a promising biomarker in digestive system tumors: a meta-analysis. *BMC Cancer*.

